# Risk factors for gambling and problem gambling: a protocol for a rapid umbrella review of systematic reviews and meta-analyses

**DOI:** 10.1186/s13643-020-01455-x

**Published:** 2020-08-27

**Authors:** Caryl Beynon, Nicola Pearce-Smith, Rachel Clark

**Affiliations:** Public Health England, Wellington House, 133-155 Waterloo Rd, London, SE1 8UG UK

**Keywords:** Umbrella review, Systematic review, Gambling, Problem gambling, Risk factors, Public health

## Abstract

**Background:**

Gambling and problem gambling are increasingly being viewed as a public health issue. European surveys have reported a high prevalence of gambling, and according to the Gambling Commission, in 2018, almost half of the general population aged 16 and over in England had participated in gambling in the 4 weeks prior to being surveyed. The potential harms associated with gambling and problem are broad, including harms to individuals, their friends and family, and society. There is a need to better understand the nature of this issue, including its risk factors. The purpose of this study is to identify and examine the risk factors associated with gambling and problem gambling.

**Methods:**

An umbrella review will be conducted, where systematic approaches will be used to identify, appraise and synthesise systematic reviews and meta-analyses of risk factors for gambling and problem gambling. The review will include systematic reviews and meta-analyses published between 2005 and 2019, in English language, focused on any population and any risk factor, and of quantitative or qualitative studies. Electronic searches will be conducted in Ovid MEDLINE, Ovid Embase, Ovid PsycInfo, NICE Evidence and SocIndex via EBSCO, and a range of websites will be searched for grey literature. Reference lists will be scanned for additional papers and experts will be contacted. Screening, quality assessment and data extraction will be conducted in duplicate, and quality assessment will be conducted using AMSTAR-2. A narrative synthesis will be used to summarise the results.

**Discussion:**

The results of this review will provide a comprehensive and up-to-date understanding of the risk factors associated with gambling and problem gambling. It will be used by Public Health England as part of a broader evidence review of gambling-related harms.

**Systematic review registration:**

PROSPERO CRD42019151520

## Background

Gambling is increasingly being identified as a public health problem [1, 2]. Harms associated with gambling are wide-ranging and include harms not only to the individual gambler but to their families and close associates as well as wider society [3, 4]. The global prevalence of problem gambling has been reported to range from 0.7 to 6.5%, and studies from across Europe have reported a high participation in gambling [5]. In 2018, a survey conducted in England by the Gambling Commission reported that almost half of the respondents had participated in gambling in the 4 weeks prior to being surveyed [6]. In addition, 0.7% of respondents were classified as ‘problem gamblers’ and an additional 1.1% of respondents were classified as ‘moderate risk’ gamblers, defined as ‘those who experience a moderate level of problems leading to some negative consequences’ [6]. The threshold for being considered a ‘problem gambler’ within this particular survey is high—a person has to score 8 or more on the Problem Gambling Severity Index (PGSI) or 3 or more according to the Diagnostic or Statistical Manual-IV [7]. So the number of people experiencing problem gambling could well be higher.

Risk factors are traits or exposures that increase the possibility that an individual will develop a condition and can be fixed or variable [8]. The risk factors for gambling and problem gambling are broad and have been reported in numerous systematic reviews and primary studies. At an individual level, risk factors include (but are not limited to) fixed biological factors, such as gender and impulsivity, and behavioural factors such as levels of participation in gambling, excessive use of alcohol and use of illicit drugs and propensity towards violent behaviour [9]. Broader factors related to the family environment [10] and gambling availability have also been identified [11]. A scoping search identified a number of systematic reviews and meta-analyses of risk factors for problem gambling, largely focused on specific risk factors or types of risk [9–11] although one focused on specific populations [12]. No systematic reviews, meta-analyses or umbrella reviews were identified examining all risk factors for all populations. In order to understand the breadth of possible risk factors driving gambling and problem gambling behaviours, there is a need to collate this review-level evidence. This work is part of a broader review examining gambling-related harms [13, 14].

The overall aim of this umbrella review is to identify the risk factors associated with gambling and problem gambling. The research questions are as follows:

1. What risk factors are associated with gambling?
2. What risk factors are associated with different levels of gambling intensity?

## Methods

This review adopted a rapid review methodology [15] to identify, appraise and synthesise systematic reviews and meta-analyses, defined here as an ‘umbrella’ review [16]. The use of existing systematic reviews and meta-analyses enables a broad examination of best available evidence in a timely way and is useful for addressing the high-level questions set out for this review, where multiple risk factors are expected to be identified. This review protocol is being reported in accordance with reporting guidance provided in the Preferred Reporting Items for Systematic Review and Meta-Analysis Protocols (PRISMA-P) statement [17] (see checklist in Additional file 1). The protocol is registered on PROSPERO (CRD42019151520). The review will be conducted using EPPI-Reviewer 4.

### Definitions of terms

There are multiple definitions of the term ‘gambling’, but for the purpose of this review, gambling is defined (as set out by the Gambling Act 2005) as *‘…*any kind of betting, gaming or playing lotteries. Gaming means taking part in games of chance for a prize (where the prize is money or money’s worth), betting involves making a bet on the outcome of sports, races, events or whether or not something is true, whose outcomes may or may not involve elements of skill but whose outcomes are uncertain and lotteries (typically) involve a payment to participate in an event in which prizes are allocated on the basis of chance.’ [4].

There is no single definition for ‘harmful’ or ‘problem’ gambling, and this can be measured in several ways. For example, reports prepared for the Gambling Commission estimate problem gambling according to scores derived from 2 different instruments: the Diagnostic and Statistical Manual of Mental Disorders IV (DSM-IV) and the Problem Gambling Severity Index (PGSI). The DSM-IV contains 10 diagnostic criteria and possible scores are between 0 and 10; a score of 3 or over indicates problem gambling. The PGSI contains 9 diagnostic criteria and a score of between 0 and 27 is possible; a score of 1–2 is ‘low risk’, 3–7 is ‘moderate risk’ and 8 and over is ‘problem gambling’ [7]. In the USA, the South Oaks Gambling Screen (SOGS) is commonly used, where positive answers to three out of twenty gambling-related questions are considered indicative of problem gambling [18]. In order to capture the breadth of literature available, no one definition will be adopted and this review will include papers which define ‘harmful’ or ‘problem’ gambling in different ways.

In the context of this review, a risk factor is defined as any factor investigated as being associated with gambling (including initiation, escalation, urge or intensity), either causally or otherwise. Where the evidence shows the link to be causal (rather than an association), this will be reported.

### Inclusion and exclusion criteria

Inclusion and exclusion criteria have been developed using an adapted version of the PICO (population, intervention, comparison, outcome) framework, as set out in Table 1.

**Table 1:** PICO-S

PICO-S component	Inclusion/exclusion criteria
Population	All populations. To include adults and children, general population or sub-groups of the population (e.g. by sex, age, ethnicity, geographical location, deprivation, institution) or a clinical population (e.g. those with Parkinson’s disease, post-traumatic stress disorder).
Issue	Risk factors associated with gambling and problem gambling. All risk factors will be included, and it is expected that they will comprise individual and clinical attributes (such as gender, impulsivity, the presence of Parkinson’s disease), as well as social and environmental factors (such as family influences, the availability of gambling). Reviews of studies examining protective factors will be excluded.
Comparison	Any or no comparisons.
Outcomes	Gambling—to include all forms of gambling, including gambling-related aspects of gaming (see definitions), different levels of gambling intensity and gambling initiation, urges or escalation.
Study type	Systematic reviews of both quantitative and qualitative studies with narrative synthesis and/or meta-analysis. Other review types (such as mapping, scoping and narrative reviews) will be excluded. Reviews of studies examining the effectiveness of interventions will also be excluded.

It is expected that two types of study will be identified for inclusion: (i) those that focus on the gambling population and explore all risk factors and (ii) those that focus on a specific risk factor.

Additional inclusion criteria:

- Language: English (other languages will not be included, due to the team’s inability to translate)
- Publication date: 1 January 2005–4 September 2019. 2005 was selected as a cut-off as in this year the Government issued proposals to reform the law on gambling [i.e. the Gambling Act] and the Economic and Social Research Council/Responsibility in Gambling Trust provided £1 million of funding for research on problem gambling—significantly increasing capacity for research on this topic in England [19].
- Publication type: peer reviewed and grey literature
- Setting: reviews of studies which are based within the Organisation for Economic Co-operation and Development (OECD). Where studies set in non-OECD countries are also included, more than half of included studies must be from OECD countries and inclusion/exclusion will be considered on a case-by-case basis.

### Search strategy

A comprehensive search will be undertaken using multiple methods to identify both published and grey literature. The search strategy was developed by a Senior Information Scientist in PHE and quality assured by a second Information Scientist.

#### Electronic searches

The following databases will be searched: Ovid MEDLINE, Ovid Embase, Ovid PsycINFO, Social Policy and Practice, Social Care Online, NICE Evidence and SocIndex via EBSCO. The number of papers retrieved from each database will be recorded. The full MEDLINE search is presented in Additional file 2; this will be adjusted for use in other databases. The search will look for terms in the title, abstract, author key words and thesaurus terms (such as MeSH Medical Subject Headings in MEDLINE) where available. The review filter will be used for all databases except for SocIndex (which does not have a validated one). For SocIndex, a set of search terms will be created in order to restrict the search to systematic reviews and meta-analyses.

#### Grey literature

Reports and other relevant literature that may not be published in databases will be sought by searching Google and websites such as those listed here (years 2005 to 2019). If a website provides a review summary, effort will be made to find the full study report.

- Gamble Aware InfoHub
- Gambling Commission
- GambLib (Gambling Research Library)
- Gam Care
- National Problem Gambling Clinic
- Gordon Moody Association
- Gamblers Anonymous
- Open Grey
- Gam-Anon
- Gambling Information Resource Office Research Library
- Advisory Board for Safer Gambling
- Gambling Watch UK
- Australian Gambling Research Centre
- Gambling Research Exchange Ontario
- Citizens Advice Bureau
- Be Gamble Aware
- Problem Gambling, Wigan Council
- Gambling Compliance
- Gambling Watch UK
- Child Family Community Australia
- International Centre for Youth Gambling Problems and High-Risk Behaviours
- Gambling and Addictions Research Centre
- Alberta Gambling Research Institute
- Responsible Gambling Council
- Problem Gambling Foundation of New Zealand
- Gambling Commission New Zealand
- Victorian Responsible Gambling Foundation

#### Handsearching

Reference lists of retrieved papers will be searched for additional relevant papers which fulfil the inclusion/exclusion criteria. In addition, if any umbrella reviews are identified, the reference lists will be scanned for inclusion.

#### Consultation with experts

Once a list of included studies is available, this will be shared with the project Expert Reference Group to check for additional studies. This group includes national and international topic experts.

### Screening and selection procedure

A pilot screen will be undertaken whereby each reviewer will independently screen the same 100 randomly selected references/papers and indicate which should be included/excluded. Reviewers will obtain the full paper if this is needed for them to make their assessment. Any discrepancies indicate inconsistencies in understanding of the inclusion/exclusion criteria between reviewers, and this stage will allow these to be identified, discussed and resolved. If necessary, the inclusion/exclusion criteria will be modified, and the changes will be recorded in a decision log.

References will be divided between four reviewers. The title/abstract of every reference will be screened independently by two reviewers (‘review pairs’) according to the inclusion/exclusion criteria, and each reference will be coded as either ‘included’ or ‘excluded’. EPPI-Reviewer will be used to measure inter-rater agreement for all reviewer pairs; agreement of 90% or over will be considered acceptable. If the agreement is less than 90%, the reason will be explored and rectified and screening will be repeated, in line with the guidance from the National Institute for Health and Care Excellence (NICE) on title/abstract screening [20].

The full articles of the remaining references will be obtained. Full articles will be divided between reviewers and screened using inclusion/exclusion codes set up in advance by the Project Team. Ten percent of the papers screened by each reviewer will be reviewed independently by a second reviewer using the ‘parent’ codes: include and exclude (i.e. rather than specific exclusion codes such as ‘date’, ‘geography’, ‘study type’). A threshold of 80% agreement will be considered acceptable in line with criteria outlined in the AMSTAR 2 (Assessing the Methodological Quality of Systematic Reviews) tool [21]. A decision on what steps should be taken if the agreement is less than 80% will be made by the Project Team should this situation arise.

### Data extraction

Data extraction tables will be used to extract the relevant information from each study. These will include the following information: authors, date, country, the PICO-S elements and the relevant results. Authors will be contacted by the reviewers to ask for missing information or clarification where necessary, and where information is considered essential. Data extraction tables will be pilot tested before being used and signed off by the Expert Reference Group. All reviewers will extract the data from a set of eligible studies; 10% of all papers will be randomly selected and the data from these will be extracted independently by a second reviewer. Agreement between reviewers for data extraction will be checked to ensure this is acceptable (at least 80%). A decision on what steps should be taken if the agreement is less than 80% will be made by the Project Team should this situation arise. The Cochrane PROGRESS-Plus tool [22] will be used to extract data on the broad dimensions of inequality.

### Quality assessment (risk of bias)

The quality of systematic reviews will be assessed using the AMSTAR2 checklist [21]. Each paper will be independently assessed by two reviewers, and disagreements will be resolved through discussion. If required, a third person will be brought in to resolve ongoing disagreements.

### Method of synthesis

Given the broad scope of this review, included studies are likely to be heterogeneous, and therefore, a narrative analysis will be conducted with text used to summarise and explain findings [23]. Studies will be summarised according to themes. An appraisal of the quality of the literature will be included. Differences by sub-group will be examined where this is reported in the literature to integrate a focus on equity, using the Cochrane PROGRESS-Plus tool [22]. The body of evidence will be assessed according to the four principles laid out in the CERQual approach which are (1) the methodological limitations of the studies which make up the evidence, (2) the relevance of findings to the review question, (3) the coherence of the findings and (4) the adequacy of data supporting the findings [24].

## Discussion

This rapid umbrella review will identify and examine the breadth of risk factors associated with gambling and problem gambling. The findings of this review will be utilised as part of a broader review of evidence conducted by Public Health England on gambling-related harms. A full report of this work will be shared and discussed with government departments and published on our government website GOV.UK. The results of this review will also be submitted for publication in a peer review journal.

Any deviations to the protocol considered necessary will be discussed by the Project Team prior to being implemented and documented in a decision log (stored in Excel) for later reporting.

A number of limitations are anticipated. The reliance on existing systematic reviews and meta-analyses is impacted by the quality of their methods and reporting—whilst we are assessing this, if the quality is poor, our ability to fully utilise their results will be limited. In addition, there may be a large number of systematic reviews and meta-analyses, and if they are focused on different risk factors, the results may be difficult to synthesise.

## Supplementary information

**Additional file 1.** PRISMA Checklist

**Additional file 2.** MEDLINE search. Full search conducted in MEDLINE, enabling replication of review

## Data Availability

Not applicable.

## Abbreviations

AMSTAR: Assessing the Methodological Quality of Systematic Reviews
CERQual: Confidence in the Evidence from Reviews of Qualitative Research
DSM: Diagnostic and Statistical Manual
NICE: National Institute for Health and Care Excellence
OECD: Organisation for Economic Co-operation and Development
PGSI: Problem Gambling Severity Index
PHE: Public Health England
PRISMA-P: Preferred Reporting Items for Systematic Review and Meta-Analysis Protocols
SOGS: South Oaks Gambling Screen

## References

[1] Gambling Commission (2018). Young People & Gambling 2018.

[2] Wardle H, Reith G, Langham E, Rogers RD (2019). Gambling and public health: we need policy action to prevent harm. BMJ.

[3] Langham E, Thorne H, Browne M, Donaldson P, Rose J, Rockloff M (2016). Understanding gambling related harm: a proposed definition, conceptual framework, and taxonomy of harms. BMC Public Health.

[4] Wardle H, Reith G, Best D, McDaid D, Platt A (2018). Measuring gambling-related harms: a framework for action.

[5] Calado F, Griffiths MD (2016). Problem gambling worldwide: an update and systematic review of empirical research (2000-2015). J Behav Addict.

[6] Gambling Commission (2019). Gambling participation in 2018: behaviour, awareness and attitudes.

[7] Conolly A, Davies B, Fuller E, Heinze N, Wardel H (2018). Gambling behaviour in Great Britain in 2016. Evidence from England, Scotland and Wales.

[8] Offord DR, Kraemer HC. Risk factors and prevention. Evid Based Ment Health. 2000;3(3):70–71.

[9] Dowling N, Suomi A, Jackson A, Lavis T, Patford J, Cockman S (2016). Problem gambling and intimate partner volence: a systematic review and meta-analysis. Trauma Violence Abuse.

[10] McComb JL, Sabiston CM (2010). Family influences on adolescent gambling behavior: a review of the literature. J Gambling Stud.

[11] Johansson A, Grant JE, Kim SW, Odlaug BL, Götestam KG (2009). Risk factors for problematic gambling: a critical literature review. J Gambling Stud.

[12] Dowling NA, Merkouris SS, Greenwood CJ, Oldenhof E, Toumbourou JW, Youssef GJ (2017). Early risk and protective factors for problem gambling: a systematic review and meta-analysis of longitudinal studies. Clinical psychology review..

[13] Public Health England (2020). Gambling-related harms evidence review: scope.

[14] Beynon C, Pearce-Smith N, Clark R (2020). Harms associated with gambling: abbreviated systematic review protocol. Systematic Reviews..

[15] Haby MM, Chapman E, Clark R, Barreto J, Reveiz L, Lavis JN (2016). What are the best methodologies for rapid reviews of the research evidence for evidence-informed decision making in health policy and practice: a rapid review. Health Research Policy and Systems..

[16] Grant MJ, Booth A (2009). A typology of reviews: an analysis of 14 review types and associated methodologies. Health Info Libr J.

[17] Moher D, Liberati A, Tetzlaff J, Altman DG, The PG (2009). Preferred Reporting Items for Systematic Reviews and Meta-Analyses: the PRISMA statement. PLoS Med.

[18] Welte JW, Barnes GM, Tidwell M-CO, Hoffman JH, Wieczorek WF (2015). Gambling and problem gambling in the United States: changes between 1999 and 2013. Journal of gambling studies..

[19] Economic and Social Research Council (2007). Annual report and accounts, 2006-07.

[20] National Institute for Health and Care Excellence (2014). Developing NICE guidelines: the manual.

[21] Shea B, Reeves B, Wells G, Thuku M, Hamel C, Moran J (2017). AMSTAR 2: a critical appraisal tool for systematic reviews that include randomised or non-randomised studies of healthcare interventions, or both. Br Med J.

[22] Cochrane Methods Group (2019). PROGRESS-Plus 2019.

[23] Popay J, Roberts H, Sowdon A, Petticrew M, Arai L, Rodgers M (2006). Guidance on the conduct of narrative synthesis in systematic reviews.

[24] Cochrane Methods Group (2019). CERQual: A new approach for supporting the use of qualitative evidence in decision making 2019.

