# Supplementary material for: Risk factors for gambling and problem gambling: a protocol for a rapid umbrella review of systematic reviews and meta-analyses
Source: Syst Rev. 2020 Aug 27;9:198. doi: 10.1186/s13643-020-01455-x (PMC7453523; doi:10.1186/s13643-020-01455-x)
Supplement: Supplementary file 2 — Additional file 2. MEDLINE search. Full search conducted in MEDLINE, enabling replication of review [file 13643_2020_1455_MOESM2_ESM.pdf]

Additional file 2: Medline Search

1. gambl*.tw,kw.
2. (Iowa adj gambl*).tw,kw.
3. 1 not 2
4. Gambling/
5. virtual good*.tw,kw.
6. (lottery or lotteries or lotto).tw,kw.
7. (scratchcard* or scratch card*).tw,kw.
8. in-game purchase*.tw,kw.
9. game credit*.tw,kw.
10. (loot box* or loot crate*).tw,kw.
11. slot machine*.tw,kw.
12. fruit machine*.tw,kw.
13. (video lottery or VLT).tw,kw.
14. casino*.tw,kw.
15. amusement arcade*.tw,kw.
16. microtransaction*.tw,kw.
17. (bingo not gene).tw,kw.
18. ((betting or bet or bets) and (horse* or racing or dog*)).tw,kw.
19. (game or games or gaming or gamer).tw,kw.
20. Video Games/
21. 19 or 20
22. (money or monetization or monetisation or monetary or reward* or win or wins or winning* or loss or losses or lose).tw,kw.
23. exp Reward/
24. 22 or 23
25. 21 and 24
26. 3 or 4 or 5 or 6 or 7 or 8 or 9 or 10 or 11 or 12 or 13 or 14 or 15 or 16 or 17 or 18 or 25
27. risk factor*.tw,kw.
28. determinant*.tw,kw.
29. (exposure or exposed).tw,kw.
30. moderator.tw,kw.

31. mediator.tw,kw.
32. hazard*.tw,kw.
33. predictor*.tw,kw.
34. indicator*.tw,kw.
35. relationship*.tw,kw.
36. association*.tw,kw.
37. vulnerabilit*.tw,kw.
38. likelihood.tw,kw.
39. susceptibilit*.tw,kw.
40. risk factors/
41. Health Status Indicators/
42. 27 or 28 or 29 or 30 or 31 or 32 or 33 or 34 or 35 or 36 or 37 or 38 or 39 or 40 or 41
43. 26 and 42
44. limit 43 to english language
45. limit 44 to yr="2005 - 2019"
46. limit 45 to "reviews (maximizes sensitivity)"
